# Role of Macrophages in Cardiac Remodeling: Cues From Zebrafish Heart

**DOI:** 10.1002/iid3.70282

**Published:** 2026-01-11

**Authors:** Himanshu Gaur, Maram Hasan, Huseyin C. Yalcin

**Affiliations:** ^1^ Biomedical Research Center, QU Health Qatar University Doha Qatar; ^2^ Department of Biomedical Sciences, College of Health Sciences, QU Health Qatar University Doha Qatar; ^3^ Department of Mechanical and Industrial Engineering Qatar University Doha Qatar

**Keywords:** cardiac repair, cardiovascular diseases, innate immune cells, macrophage polarization, zebrafish

## Abstract

**Background:**

Macrophages are a key component of innate immunity and regulate cardiac phenotypes by their polarization state. The classical M1 macrophages are activated by pro‐inflammatory stimuli, whereas M2 macrophages are activated by anti‐inflammatory stimuli. The balance between M1 and M2 polarization is important and tightly controlled to maintain tissue homeostasis.

**Objective:**

This review aims to explain the diversity of the ability to regenerate among humans and zebrafish, the role of the immune system in heart regeneration, and macrophage function in normal conditions and disease. It also investigates age‐related effects on macrophage function and therapeutic strategies to manipulate macrophage polarization in the treatment of heart injury.

**Methods:**

Systematic review of the literature was conducted focusing on macrophage polarization, cardiac regeneration mechanisms, and immunomodulatory therapy.

**Results:**

Macrophage polarization imbalances of M1‐M2 are involved in inflammatory and cardiac disease. Mechanisms of macrophage function under various states and in various species are useful for insightful comprehension of novel therapy strategies. Macrophage polarization modulation is a future potential strategy for cardiac repair and the treatment of cardiac disease.

**Conclusion:**

Targeting macrophage polarization to restore balance and homeostasis is a promising strategy for supporting cardiac regeneration and the treatment of inflammatory cardiac disease.

## Summary

- We summarized the role of macrophages in cardiac remodeling.
- Macrophage polarization and their microenvironment g the fate of cardiac remodeling.
- Different regenerative capabilities, and how they are influenced by various factors such as age are explained.
- Possible therapeutic approaches for changing macrophage polarization to augment cardiac repair are highlighted.

## Graphical abstract

The figure illustrates the role of macrophage polarization in cardiac damage and repair, highlighting monocyte to M0, M1, and M2 macrophage differentiation. M1 macrophages, in response to pro‐inflammatory cytokines (e.g., IFNγ, TNF‐α, IL‐6), are involved in fibrosis and scarring in the heart. M2 macrophages, in response to anti‐inflammatory cytokines (e.g., IL‐10, TGFβ), are involved in tissue restoration and regeneration. Various macrophage polarization modulating approaches to reverse scarring and support repair, such as microRNA modulation (e.g., miRNA‐98, miRNA‐21), chemical agents (e.g., Poly (I:C), Dapagliflozin), membrane potential manipulation (e.g., Glibenclamide), direct cardiac cell fate reprogramming, and cell‐based immunotherapies (e.g., CAR‐M and direct transplantation), are also highlighted.

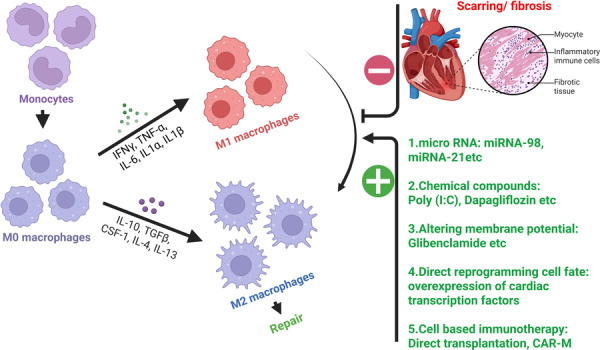

## 1 Introduction

Cardiovascular diseases (CVDs), the disease of the heart and blood vessels, are the major cause of death and disability and are estimated to claim 23.6 million deaths globally in 2030 [1]. Myocardial infarction (MI) and heart failure (HF) are the major cause of deaths through CVDs. MI is due to the occlusion or limited supply of blood to an area of the heart muscle [2] and can be detected by a number of biomarkers such as cardiac troponin I (cTnI), myoglobin (MYO), creatine kinase MB (CK‐MB), and B‐type natriuretic peptide (BNP) [3]. Even so, due to widespread myocardial damage, muscles in the heart cannot pump blood and therefore inadequate cardiac output leads to an another medical condition, HF [4]. HF is the most serious health status in which the heart's ability to pump blood to meet the body's oxygen and nutrient needs are decreased [5]. HF can also be classified into systolic and diastolic forms based on the ventricular functioning capacity for ejecting or receiving blood [6]. Systolic HF is such a type wherein the condition of the heart as a contractor becomes compromised, causing reduced cardiac output, and it is known as HF with reduced ejection fraction (HFrEF). On the other hand, diastolic HF is characterized by the occurrence of abnormalities in heart relaxation and stiffness, affecting the filling of the heart with blood properly, and referred to as HF with preserved ejection fraction (HFpEF) [6, 7]. Patients with HF have a high incidence of complications, like arrhythmias, which may result in sudden cardiac medical conditions [8]. Despite the existence of advanced treatment facilities, MI frequently culminates in HF, and is one of the main causes of death in the developed world [9].

Since loss of cardiac muscle is due to MI and HF, regeneration of lost tissue would be beneficial for recovery. Therefore, regeneration of lost cardiac muscles remains a topic of interest, to treat the most prevalent CVD disorders and maintain the integrity of the heart [10]. The significance of cardiac regeneration lies in its potential to ease the heavy heart diseases burden through the return of the function of the heart and thereby improving results for patients [11]. Unfortunately, the human heart loses its regeneration potential to an incredibly basal level, not enough for rescuing the substantial cardiac insult on aging. In fact, human heart is one of the least regenerative organs in the body and replaces infarcted myocardium with non‐contractile scar tissues instead of new muscles [12]. This is because of limited cardiomyocyte proliferation [12]. On the contrary, various organisms like zebrafish, axolotl, and newts regenerate their heart following significant cardiac damage [13]. Therefore, to better understand the human heart regeneration and new methods thereto, one has to investigate the cardiac regeneration in the lower vertebrates. Zebrafish is one of the most used model organisms for this purpose.

Inflammation is a key part of tissue regeneration and also plays role in tissue remodeling in general. For cardiac regeneration, at an immunological level, inflammation helps to regulate the progression and/or repair of diseased tissue [14]. In this process, damaged cardiac cells (epithelial and endothelial cells) trigger inflammatory cascade that recruits immune cells such as neutrophils and monocytes through chemokines and growth factors which then differentiate monocytes in the scar tissue into macrophages [14, 15, 16]. Macrophages are ubiquitous and comprise diverse cell types with different phenotypes and functions. Today it is well known that macrophages are key immune effector cells and perform vital homeostatic functions. Furthermore, macrophages play critical roles in the development, tissue remodeling, and are essential for disease advancement [17]. Macrophages play a crucial role in disease development and healing, by its dual‐functionality (pro‐inflammatory and anti‐inflammatory) [18] based on micro‐environmental signals [19]. Macrophages play a critical role in the function of innate immunity by the process of phagocytosis [20]. Macrophage phagocytic receptors recognize apoptotic neutrophils and modulate the number of neutrophils in inflammation, supporting neutrophils that are highly crucial in cardiac repair and regeneration [21]. In zebrafish, macrophages stimulate the epicardium that again triggers the cardiomyocyte proliferation, eventually giving rise to cardiac regeneration [22]. It is important, along the way, macrophages change their morphology and functions, and are further divided into M1 and M2 macrophages [18]. M1 macrophages are classically activated macrophages, and are classified as pro‐inflammatory macrophages. They are triggered by pathogen associated molecular patterns (PAMPs) such as lipopolysaccharides and with cytokines such as interferon‐γ (IFN‐γ). The activated M1 macrophages trigger further the production of tumor necrosis factor‐α (TNF‐α) and pro‐inflammatory cytokines such as interleukin (IL)−1α, IL‐1β, and IL‐6 [23, 24]. M2 macrophages are termed alternatively activated macrophages and known as anti‐inflammatory macrophages. M2 macrophages are activated by cytokines like colony stimulating factor‐1 (CSF‐1), transforming growth factor‐β (TGF‐β), IL‐4, IL‐10, and IL‐13 [24, 25]. The activated macrophages function differently under pathological conditions whereas under normal physiological conditions, they have homeostasis. For instance, in cancer, M1 macrophages exhibit tumoricidal functions, while M2‐like macrophages promote tumor progression and metastasis [26] whereas in CVDs M1 macrophages exhibit CVDs progression and prolonged inflammation while M2 macrophages are involved in repair and healing processes [26, 27]. More importantly, phenotypic conversion of M1 pro‐inflammatory to M2 anti‐inflammatory phenotype is crucial for successful repair or regeneration process. Though still molecular pathways for such processes are poorly defined [28]. Therapeutically, one evidence revealed that, administration of neonatal cardiac macrophages augments adult heart restoration and stimulates increased adult cardiomyocyte proliferation [29] illustrating clinical potential of macrophage interventions in curing CVDs.

Thus, increasing interest exists in the investigation of the function of macrophages in cardiac inflammation and repair. Due to its popularity as an in vivo model, Zebrafish is suitable organism for investigation of the relevant mechanisms. This review in current era gathers the role of macrophages in the zebrafish heart regenerative process and evokes probable paths of the improvement in regenerating strategy in human cardiac muscle regeneration.

### 1.1 Heart Regeneration in Human and Zebrafish

**Figure 1:**
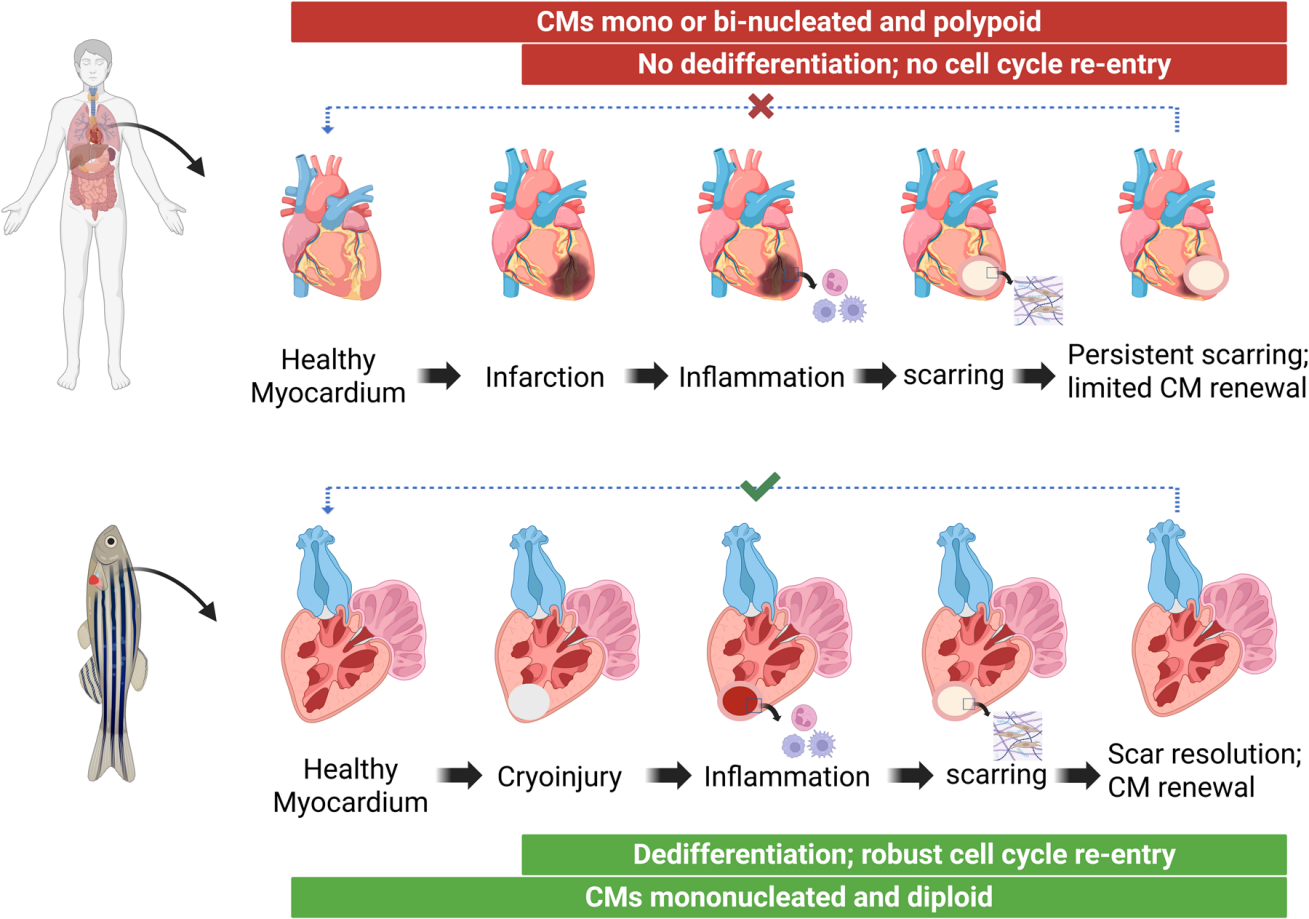
This illustration demonstrates contrasting features of cardiac regeneration in humans and zebrafish following damage. Human cardiomyocytes (CMs) are mono‐ or bi‐nucleated and polypoid, and they do not dedifferentiate or re‐enter the cell cycle in MI patients. Importantly, renewal of cardiomyocytes is low and leads to chronic scarring and fibrosis. In contrast, zebrafish CMs are diploid and mononucleated, and can dedifferentiate and re‐enter the cell cycle following cardiac injury including cryoinjury (indicated). The zebrafish regenerative capacity allows, to resorb scar tissue and regenerate functional cardiomyocytes, illustrating a clear difference in regenerative capacity between the two species.

Cardiac regeneration depends mainly on the proliferative capacities of the cardiomyocytes. The adult humans show negligible or limited cardiac regeneration potential whereas adult zebrafish hearts regenerate robustly. The CM proliferative capacity varies significantly between humans and zebrafish which is probably influenced by genetic, developmental and environmental factors. Understanding these differences can potentially provide insights into disease control and regeneration. Zebrafish potentially demonstrate CM dedifferentiation and proliferation following the injury helping in restoring the cardiac muscle following injuries [30, 31]. Zebrafish CMs are mononucleated and diploid [32] and performs robust cell cycle re‐entry, allowing zebrafish to complete the regeneration process [30]. Whereas, in humans, CMs are mono‐or bi‐nucleated with polyploidy status and they lost their capacity of proliferation after few months of birth when CM exit from the cell cycle re‐entry process and becomes terminally differentiated (Figure 1) [33, 34]. Zebrafish, unlike humans, possess a wonderful regenerative capacity, which makes them capable of fully regenerating their hearts upon severe injuries such as ventricular resection and cryoinjury [35, 36, 37, 38]. This is brought about by cardiomyocyte proliferation, a vital process for zebrafish heart regeneration [36]. Unlike this, however, the mammalian adult heart lacks a robust regeneration capability following damage, and rather, becomes substituted by non‐contractile scarring and recovery in part [36, 39]. Humans, in the state of high blood pressure, deposits a thick collagen layer to strengthen the ventricular wall, leading hearts to develop an aversion non‐contractile scar over time while zebrafish forms a thin, loose collagen‐rich scar, leading the scar to resolve efficiently [40].

**Figure 2:**
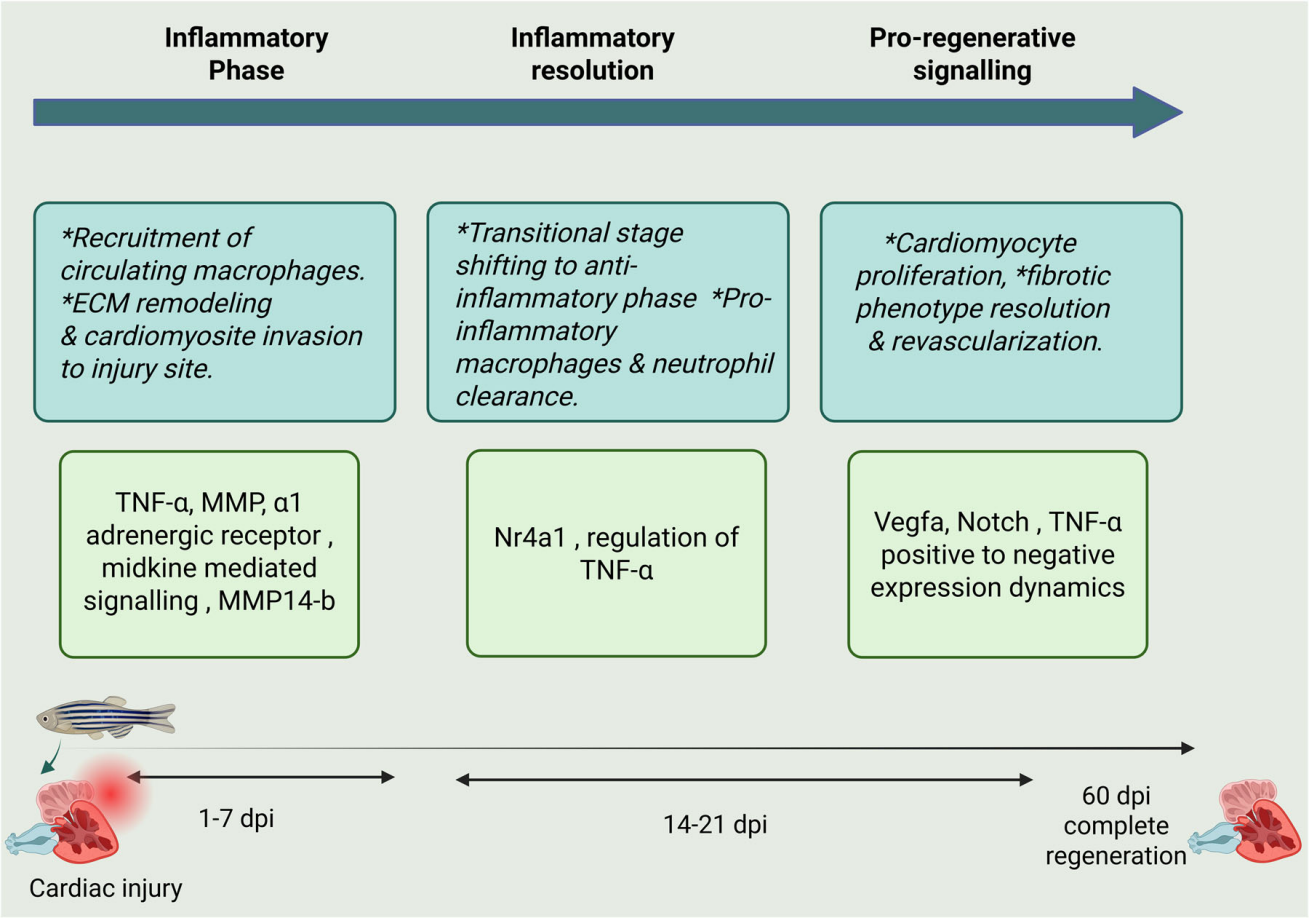
This illustration demonstrates zebrafish cardiac regeneration processes, with few key molecular pathways involved in the inflammatory phase, inflammatory resolution, and pro‐regenerative signaling. The inflammatory phase (1–7 days post‐injury, dpi) is characterized by invasion of circulating macrophages and ECM remodeling, which are mediated through TNF‐α, MMP, α1 adrenergic receptor signaling, and midkine‐mediated signaling. The inflammatory resolution phase (14–21 dpi) turns anti‐inflammatory processes, with regulation by Nr4a1 and elimination of pro‐inflammatory macrophages and neutrophils. Finally, during the pro‐regenerative signaling phase (60 dpi), signals like VEGFA, Notch, and TNF‐α activate cardiomyocyte proliferation, fibrotic phenotype resolution, and revascularization, leading to complete cardiac regeneration.

Macrophages are very crucial in both species with differing capacities. In zebrafish, they promote CMs proliferation and revascularization and thus regeneration process [41] whereas in humans, macrophages role often leads to fibrosis and permanent scarring post‐injury [42, 43]. Zebrafish demonstrated impaired cardiac tissue regeneration and neovascularization when macrophage recruitment was experimentally overdue in cryo‐injured hearts [41]. It is proven that the genetically engineered and optically accessible zebrafish larvae serve as a good and reliable experimental model for studying macrophage polarization in living settings, as it demonstrated the similarity between the M1 and M2 macrophage subtypes found in zebrafish and those in mammals [44, 45]. This whole process of regeneration is regulated by critical pathways briefly described in (Figure 2) mediated by TNF‐α, matrix metalloproteinases (MMPs), Nuclear receptor 4A1 (nr4a), Vascular endothelial growth factor A (vegfa) etc [12, 46, 47, 48, 49].

### 1.2 Involvement of the Immune System in Heart Regeneration

The immune system is critically important during heart regeneration process. Innate and adaptive immune response is responsible in the regeneration of heart after damage, infection or injury. Mast cells, neutrophils, monocytes and macrophages are innate immune cells and T lymphocytes and B lymphocytes are cells responsible for adaptive immune response [50]. Pro‐inflammatory mediators such as TNF‐α, histamine and resident cardiac mast cell proteases from resident cardiac mast cells, help in triggering the cascade of signaling involving adjacent resident macrophages, endothelial cells and later on infiltrating neutrophils [51]. Neutrophils are considered as early responders to the injury after receiving signals from resident cardiac cells like mast cells [52]. Neutrophils populate to the injury within a day in human and initiate an inflammatory reaction helping in repair process [53]. Importantly, timely neutrophil clearance is required because neutrophils generate reactive oxygen species (ROS) and secrete proteases, which further degrades extracellular matrix (ECM) and worsen the post‐injury inflammatory responses [54, 55]. In zebrafish, neutrophils infiltration has been observed to increase as early as 6 h to a peak at 2 days post injury and drops down to 7 days [41]. Moreover, pro‐inflammatory neutrophils undergo apoptosis via efferocytosis and are eliminated with anti‐inflammatory monocytes or macrophages [56]. The efferocytosis and macrophage polarization are closely linked. When macrophages cleared the damaged cells, they shift the polarization towards M2 type. This transition is facilitated by the release of anti‐inflammatory signals including cytokines and growth factors that promote repair and limit excessive inflammation [57]. Fate tracing of tnfa+ macrophage in zebrafish infection model demonstrated that M1 macrophages converted to M2‐ like phenotype during resolution step suggesting proper efferocytosis [44]. If efferocytosis get impaired then the process of proper repair hampered drastically. Mycobacterium infections in zebrafish significantly disrupt macrophage at wound sites, impair their activation and alter polarization. This dysregulation overall impairs the regenerative process as the infectivity of macrophages is increased to clear apoptosis cells [58].

Monocytes or macrophages are the prevalent cell types that infiltrate the injured myocardium [59]. CCR2 (a chemokine receptor) defines the migration of the monocyte population, as CCR2^‐^ are resident cardiac macrophages whereas CCR2^+^ are circulating macrophages [60]. During injury, CCR2^‐^ macrophages decrease in number but CCR2^+^ macrophages expressing surface expressed glycoprotein lymphocyte antigen 6 C (Ly6C) are populated to the site of injury after the expression of CCL2 (a chemokine ligand) by resident macrophages and cardiomyocytes [50, 61, 62].

CCR2^+^Ly6C^+^ monocytes, macrophages differentiated from them, first differentiate into CCR2^+^Ly6Chigh macrophages (a pro‐inflammatory sub‐population) [63], and subsequently phagocytose neutrophils [64], and subsequently enhance anti‐inflammatory and pro‐fibrotic cytokines like IL10 and TGFβ [65]. With these cues, CCR2^+^Ly6Chigh macrophages become differentiated to CCR2^+^Ly6Clow macrophages in promoting fibrotic cardiac repair [66]. CCR2‐ccl2 is implicated in zebrafish inflammatory response, while the CCR2 inhibitors in zebrafish, significantly influence the movement of macrophage to the inflammatory site suggesting their presence is crucial for the movement of macrophage in response to chemotactic signals such as ccl2 stimulation. Results from Morpholino knockdown studies revealed that the full length of CCR2 is essential for proper macrophage functioning and further repair process [67, 68].

Also, upon activation by antigen presenting cells (APCs), adaptive immune T‐cells‐ specifically the circulating CD4^+^ cells‐ migrate to the injury site and augment the repair process [69] suggesting CCR2^+^Ly6Clow based repair mechanism. In contrast, B‐cells assist the infiltration of circulating CCR2^+^Ly6Chigh monocytes, which subsequently supplement tissue damage [70].

### 1.3 Involvement of Macrophages in Heart Regeneration

#### 1.3.1 Macrophages Under Normal vs Diseased Conditions

Macrophages function to preserve the healthy state of the tissues for normal homeostasis considered as normal physiological states whereas under the condition when these are not fulfilled then pathological or diseased symptoms are exhibited by the body. Macrophages maintain the overall homeostasis through removal and recycling of apoptotic cells, and tissue debris through the process of phagocytosis [71] and thus inhibiting the accumulation in the organs while macrophages that are unsuccessful in the removal of debris and inhibition of accumulation greatly impair the functioning of organs [72]. Apart from phagocytic function, non‐phagocytic function is also pivotal in heart regeneration following an injury which is one of the key functions to regulate the inflammatory environment post injury. Macrophages release various factors for facilitating communication among cells. Specifically, cytokines and growth factors secreted by macrophages help in cardiomyocyte proliferation and angiogenesis [73]. Additionally, they influence ECM remodeling and fibroblast activation, which is significant in new tissue formation post injury [74, 75].

In zebrafish, *mpeg1.1* ^*+*^ (mpeg:1.1 promoter derive expression in macrophage) macrophages are present in uninjured tissue for the maintenance of homeostasis while the size and number of *mpeg1.1* ^*+*^ cells increase post cardiac injury suggesting that the injured heart is approaching homeostasis [76]. It is important to note that macrophage M1 and M2 subtypes balance or phenotypic switching is very crucial for maintaining tissue homeostasis, otherwise an imbalance of M1 and M2 macrophages will promote inflammatory diseases like atherosclerosis [77]. For instance, during zebrafish post cardiac injury, csf1ra promotes early pro‐inflammatory response and scarring while subsequent macrophage phenotypic switch to anti‐inflammatory state allows scar resolution and ultimately maintains the organ homeostasis. This phenotypic switching was reported to reduce the tNF‐α and increase spp1 expression levels. Interestingly, the tNF‐α^+^ pro‐inflammatory macrophages promote scar deposition whereas tNF‐α^‐^ macrophages support scar resolution [78].

#### 1.3.2 Age Dependent Role of Macrophages

Aging has a significant influence on functionality and responses of macrophages, moreover, additional investigations are required for understanding therapeutics for older people [79]. Researchers have shown that, macrophages from aged mice can exhibit a pro‐inflammatory role, cholesterol biosynthesis dysregulation, and insulin resistance [80]. Additionally, to study impaired macrophage functions such as phagocytosis and NLRP3 inflammasome‐ mediated innate immune responses, advanced glycation end products (AGEs) has been observed [81, 82]. AGEs further supported the differentiation of macrophage to the pro‐inflammatory M1 phenotype through RAGE/NF‐κB pathway [83]. Aging also affects the inflammatory processes, and low grade chronic inflammation or inflammaging is a hallmark of aging. Aging depletes tissue resident macrophage and hampers the repair process [84]. Importantly, aging changes the morphology of macrophages to an irregular shape, characterized by elongated and larger size [85, 86]. It is interesting that, studies on Csf1r zebrafish mutant showed that macrophages retain other non‐phagocytic functions. Juvenile zebrafish of this mutant line exhibits systemic macrophage depletion [87]. In another zebrafish study, it is observed that aging affects the cardiac repair process through accumulation of collagen and immune cells including macrophages and thus disturbing the homeostasis process [88]. Aging also affects glycolysis and mitochondrial oxidative phosphorylation and allows impairment in macrophage function by creating an energy deficient environment [89]. In contrast, zebrafish studies suggested that glycolysis help in border zone cardiomyocyte proliferation and cardiac repair through Nrg1/ErbB2 signaling [90].

#### 1.3.3 Cell Type Specific Macrophages

Tissue‐resident macrophages are strategically positioned in various tissues to maintain the homeostasis and act as an important part of the immune system through immune surveillance and response against infections or injuries [91]. These macrophages are heterogeneous in nature with distinct functions and are believed to originate from embryonic precursors and hematopoietic stem cells [92, 93]. Likewise cardiac tissue resident macrophages perform similar functions such as cardiac homeostasis, and response to injury, to promote tissue repair. Cardiac resident macrophages are also heterogenous and can be maintained through local proliferation and recruitment [94]. Following injuries, they engage themselves in clearing necrotic cells, reducing inflammation, promoting angiogenesis, and limiting damage extension [95]. The cardiac macrophage populations share a distinct ontology including yolk‐sac derived, fetal monocyte derived and adult monocyte derived [73]. Fate mapping suggested that different populations co‐exist at homeostasis [96]. In embryogenesis, the majority of the macrophages are derived from yolk‐sac [97] and establish themselves to perform organogenesis including angiogenesis and extracellular matrix remodeling. Yolk sac derived cells enrich the heart which is crucial for early development [98]. As development progresses, the yolk sac derived macrophages shift to fetal derived macrophages. This transition maintains the cellular diversity of macrophages, assuring proper immune responses and tissue homeostasis at later stages of development [99]. In response to cardiac injury, resident macrophages transition from a homeostatic to inflammatory type by releasing cytokines and thus promote tissue remodeling [100]. Timely activation and recruitment of macrophages to the injury site is pre‐requisite to facilitate proper repair process [101]. In regenerative zebrafish heart on the other hand, single cell analyses suggested that majority of the cardiac resident macrophages (hbaa^+^ macrophages and timp4.3^+^ macrophages) are involved in the repair process following injury [102].

#### 1.3.4 Single Cell Analyses Derived Cardiac Macrophages Subtypes and Functions

Many studies confirmed the remarkable role of cardiac resident macrophages in tissue regeneration and repair post‐injury in zebrafish embryos. In one study, authors have analyzed the cardiac resident macrophages in different conditions of the zebrafish heart model, including the uninjured (naïve), regenerative cryoinjured, and nonregenerative injured heart models. It was demonstrated that there are subsets of cardiac resident macrophages: Mac 2, Mac 3, representing the dominant cluster in the naïve heart model, Mac 1, Mac 4, Mac 8, and proliferative Mac 5, indicating the self‐regenerative nature of this cluster. While in the case of the injury of the regenerative model, it was confirmed that it notably showed continuous proliferative activity throughout 7days post cardiac injury, on the other hand, Mac 1, Mac 4, and Mac 5 showed a quick surge after 1 day of cryoinjury, and by the 7th day their level reached the reference point. This leads to the conclusion that the Mac2 and Mac3 subsets are the primary contributors to zebrafish cardiac tissue regeneration [102]. Resident macrophages efficiently support the resolution of the cardiac insult and establish the base of effective tissue regeneration via their characteristic quick response and recruitment to the site of cardiac injury before the circulating macrophages reach the site [102, 103]. Moreover, resident macrophages are essential and are required for resolving cardiac tissue fibrosis and inflammation, as well as regulating revascularization in the cardiac regeneration process. It was shown that circulating macrophages can neither substitute nor do the job of the resident macrophages in cardiac regeneration, as the cardiac regeneration process can only be elicited by the resident subpopulation [102].

Zebrafish heart resident macrophages have a low pro‐inflammatory phenotype, are more angiogenic, and hence these are less involved in fibrosis when compared to the recruited macrophages. Eventually, both types of macrophages need to collaborate in their own way to achieve successful and efficient cardiac tissue regeneration [104, 105].

### 1.4 Cardiac Repair Strategies Through Macrophage Polarization

Following a myocardial infarction, the repair process involves dedifferentiation and proliferation of cardiomyocytes. The repair process can be achieved through modulating innate immune response. Current understanding suggests that modulation of macrophage polarization might act as a potential therapeutic target in treating cardiac injury [106]. microRNAs are among the candidate molecules since these are small noncoding RNA, and were reported to modulate macrophage activity in pro‐inflammatory M1 type, or anti‐inflammatory M2 type [107]. microRNA‐98 and microRNA‐21 were reported to promote regulation of activation of IL‐10 based anti‐inflammatory macrophages and monocytes polarization [108, 109, 110] whereas inhibition of microRNA‐494‐3p, MicroRNA‐216a, and miR‐27b were reported to regulate the macrophage polarization and help reducing atherosclerotic plaques [111, 112, 113].

Other chemical drug inhibitors or activators have also been reported to influence macrophage function and polarization [111]. TLR agonist lipopolysaccharide (LPS) also resolved the inflammation and induces efferocytosis by macrophages [114]. Likewise, another TLR agonist Poly(I:C), in non‐regenerated medaka model were shown to result in the recruitment of macrophages to the site of injury and helps in the repair process through CM proliferation and scar resolution [41]. The therapy of interleukin‐4 (IL‐4), a potent regulator of immunity, were observed to regulate the macrophage polarization towards anti‐inflammatory M2 type by increasing their number following injury [115, 116]. A low molecular weight, non‐peptide Angiotensin 1‐7 (Ang‐(1‐7)) mimetics AVE0991 with its anti‐atherosclerotic and anti‐inflammatory effects suppresses the pro‐inflammatory M1 macrophage differentiation [117]. A caspase‐1 inhibitor VX765 enhances IL‐4 induced M2 polarization and importantly, promotes increased expression of M2 macrophage marker mannose receptor 1 and arginase 1 [118]. Cholesterol lowering drug Rosuvastatin is reported to exert anti‐atherosclerotic effects through macrophage polarization towards anti‐ inflammatory M2 type. This polarization is governed by increased M2 macrophage markers arginase‐1 and CD206 and decreased M1 pro‐inflammatory marker inducible nitric oxide synthase (iNOS) expression. Rosuvastatin works through inhibiting the PI3K/Akt/mTOR signaling pathway [119]. A widely used type‐2 diabetes drug Sitagliptin, attenuates early atherosclerosis by promoting M2‐polarization via chemokine stromal cell‐derived factor‐1/C‐X‐C chemokine receptor type 4 (SDF‐1/CXCR4) signaling [120]. Similarly, Dapagliflozin, a new class of antidiabetic drugs, sodium‐glucose co‐transporter 2 (SGLT2) inhibitors, polarizes macrophages towards M2 type and exerts anti‐inflammatory effects irrespective of glucose concentrations. Importantly, expression level of anti‐inflammatory miR‐146a is increased under this exposure while it was shown to decrease for pro‐inflammatory miR‐155 [121]. Likewise, a number of other chemicals or substances promotes polarization towards M2 macrophage type including Curcumin, Prostaglandin E2, Melatonin, Protocatechuic acid (PCA), Ginsenoside Rb1, Crocin [111]. Very interestingly, altering the membrane potential also changes the macrophage polarization status, for instance, blocking of K_ATP_ channel by Glibenclamide changes macrophage polarization towards M2 type through enhancing M2 marker CD206 [122].

Yet another approach for macrophage recruitment and polarization is through direct reprogramming of cell fate, such as through overexpression of cardiac transcription factors Gata4, Mef2c, and Tbx5 (GMT), GMT plus Hand2 (GHMT), or Mef2c, Myocd, and Tbx5 significantly converted fibroblasts to cardiomyocyte‐like cells [123]. Direct transplantation of neonatal cardiac macrophages also significantly rescues the infarcted area and promote heart regeneration [29].

Macrophage‐cell based immunotherapy is a promising area of study, Wang et al. 2024, demonstrated that chimeric antigen receptor‐ macrophage (CAR‐M) decreases myocardial ischemia‐ reperfusion injury. This landmark study may open up the cardiovascular related therapeutic methods such as for fibrotic phenotype, which is a major problem concerning cardiac illness in humans [124].

### 1.5 Future Directions

When different aspects of macrophages are realized, various questions remain unanswered: Macrophage polarization were identified as an important although cardiac repair mechanism, from multiple studies mainly on mammalian mice model. Mice possesses fibrotic pattern following injury while zebrafish has advantage of resolution of scar or fibrosis, which would help to realize function of macrophage polarization for clearance of fibrosis. A number of natural or synthetic substances help in macrophage polarization, and these can be studied in zebrafish to find out how do they make the microenvironment ready for polarization or whether their binding change the phenotype of polarization irrespective of microenvironment? Eventually, allogenic or autogenic macrophage transplant will be the next treatment therapy, although it is also followed by its own limitations like maintaining microenvironment favorable for needed macrophage phenotype. Particularly, it is expected that engineered macrophage will have substantial success owing to personalization.

## Author Contributions

**Himanshu Gaur:** conceptualization (lead), writing – original draft (lead), writing – review and editing (lead), validation and illustration (lead). **Huseyin C. Yalcin:** conceptualization (equal), writing – review and editing (supporting), validation (equal), supervision (lead), project administration and resources (lead). **Maram Hasan:** writing – review and editing (supporting).

## Ethics Statement

The authors have nothing to report.

## Consent

The authors have nothing to report.

## Conflicts of Interest

The authors declare no conflicts of interest.

## Data Availability

The authors have nothing to report.
